# Early Sexual Initiation Is Associated with Suicide Attempts among Chinese Young People

**DOI:** 10.3390/ijerph19073966

**Published:** 2022-03-26

**Authors:** Jianing Ren, Xinran Qi, Wenzhen Cao, Zhicheng Wang, Yueping Guo, Junjian Gaoshan, Xiao Liang, Kun Tang

**Affiliations:** 1 Vanke School of Public Health, Tsinghua University, Beijing 100084, China; 2111210093@stu.pku.edu.cn (J.R.); qixinran@ccmu.edu.cn (X.Q.); wangzc0601@tsinghua.edu.cn (Z.W.); 2 School of Public Health, Peking University, Beijing 100191, China; 3 School of Nursing, Capital Medical University, Beijing 100069, China; 4 School of Public Health and Management, Wenzhou Medical University, Wenzhou 325035, China; caowz@pku.edu.cn; 5 Research Center for Public Health, School of Medicine, Tsinghua University, Beijing 100084, China; 6 School of Journalism and Communication, Graduate School of Chinese Academy of Social Sciences, Beijing 102488, China; yueping.guo@savethechildren.org; 7 Department for Health, University of Bath, Bath BA2 7AY, UK; j.gaoshan@gmail.com; 8 China Family Planning Association, Beijing 100035, China; cfpa_lx@163.com

**Keywords:** early sexual initiation, early sexual debut, suicide attempts, young people, Chinese

## Abstract

This study aimed to investigate the association between early sexual initiation and suicide attempts (SAs) among Chinese young people. Our analysis included 9131 college students who had sexual experience from a national sample of 31 provincial administrative regions. Self-reported age at first intercourse was categorized as ≤15, 15–18, and ≥18 years, and the experience of SAs was recorded and analyzed. Compared with females whose sexual debut age was ≥18 years, those ≤15 years (defined as early sexual initiation) had higher odds of SAs in both the forced debut group (odds ratio (OR) 17.04, 95% confidence interval (CI) 4.87–59.66) and the voluntary debut group (OR 37.63, 95% CI 14.96–94.66). Early sexual initiators who lived in rural areas were more inclined to have SAs (female: OR 65.76, 95% CI 19.80–218.42; male: OR 15.39, 95% CI 1.64–144.19). Early sexual initiators who never had parent–child communication about sex were more likely to report having SAs (female: OR 37.81, 95% CI 12.28–116.46). Sexual debut during adolescence, particularly early sexual initiation, was a crucial risk factor for SAs among both sexes. Comprehensive sexuality education and smooth parental communication about sex will provide a supportive environment for young people and hence reduce the potential risks of SAs.

## 1. Introduction

In recent decades, the average age at sexual debut has declined, and sexual activity before age 15 has become more common in most Western countries [1,2]. A similar trend has been seen in Chinese young people. A report in 2000 indicated that the peak age of sexual initiation was 3 years younger than that of the previous decade [3]. Early sexual initiation, typically defined as sexual debut occurring at or before 15 years of age [4,5,6], has been correlated with a variety of sexual risk factors, such as multiple sexual partners, unintended pregnancy, and higher rates of sexually transmitted infections [7,8,9]. Besides, it also brings heavy pressure and burdens, which can produce a series of negative moods and cause the formulation of suicidal ideation and attempts [10].

Suicide is a leading cause of death worldwide among young people, with the prevalence of suicidal ideation and behaviors increasing dramatically during adolescence, a unique period of developmental vulnerability for suicide [11]. In a study with 229,129 adolescents from 59 low-income and middle-income countries, the incidence of suicidal ideation, suicide planning, and SAs was approximately 17% [12]. Another study found that the prevalence of suicidal ideation in the past year was 7.5% in Chinese college students [13]. Many risk factors have been identified for youth suicidal behaviors [14,15], and sexual experience appears to be one of the factors relating to increased risks of SAs among adolescents [16,17,18,19]. However, the association between early sexual initiation and SAs has not been explored in Chinese young people.

A possible explanation for this relationship is that young people at an early age may suffer from the adverse mental results caused by forced sexual debut, especially in the stage when they are incapable of making rational decisions or controlling emotional impulses [20,21,22], which consequently formulates SAs. Some studies found that having suicidal ideation or behaviors was correlated with forced sexual intercourse experience, a traumatic life event that negatively influences psychological health and leads to suicidal thoughts [23,24]. However, it remains unclear and needs to be explored whether the same result exists in the relationship between voluntary sexual debut and SAs. 

For young sexual initiators, traditional social norms towards sex may also contribute to suicidal behaviors. It is of special significance to study this issue in China since early sexual intercourse is unacceptable in Chinese society. Students who reported that they were in favor of teenage sex appeared to be in the minority, ranging from 26.2% of junior to 34% of senior high school respondents [25,26]. Thus, those who had an early sexual debut may be more likely to feel excluded and have a lack of social support because of the sexual conservatism towards adolescent sexuality, which lead to their suicidal thoughts. Social norms influence adolescent sexual attitudes through large environments such as area of residence and small environments such as family.

Attitudes regarding sex in rural areas are generally considered to be more conservative compared to those in urban areas. According to one study, rural adolescents were more likely to report experiencing sexual guilt and possessing more conventional, conservative gender and sexual views, for example, the sexual double standard [27]. In China, there also remains a general reluctance for parents to openly discuss sex with their children. However, evidence showed that having parent–adolescent communication about sex could effectively reduce adolescent engagement in sex [28], which may also prevent the adverse outcomes of suicide. Therefore, the area of residence and frequency of parent–child communication about sex, to some extent, indicate sexual conservatism in China, which should be considered in the complex association between early sexual initiation and SAs.

So far, there is limited literature examining the relationship between age at sexual debut and SAs [10,29,30,31,32]. The existing studies have lacked the classification of forced or voluntary sexual initiation and have not considered the impact of Chinese social norms towards sex. To resolve these limitations of previous studies, we sought to assess whether there was an association between early sexual initiation and SAs and to quantify any such relationship among young people who had ever had sexual intercourse using a national sample of Chinese college students. Examination of this association would allow a better understanding and prevention of the suicide risk of young people initiating early sex. The study hypotheses were: (1) Early sexual initiation would be associated with a higher risk of SAs; (2) The extent of the relationship between early sexual debut and SAs would be different when considering the classification of forced or voluntary sexual initiation and the sociocultural background towards youth sexuality.

## 2. Methods

### 2.1. Study Design and Participants

Data from the National College Student Survey on Sexual and Reproductive Health (NCSS-SRH) 2019, which included 31 provincial administrative regions in mainland China, were analyzed. NCSS-SRH used multi-stage sampling to select 241 sample higher education institutions (HEIs) across China. Based on the list of HEIs announced by the Ministry of Education of China, school region (eastern, central, and western) in the first sampling stage and school type (vocational college and university) in the second stage were balanced proportionally. This national survey was commissioned by the China Family Planning Association. The data collection was carried out by the China Youth Network, the largest Chinese national voluntary organization of peer educators on sexual and reproductive health.

Concerning better personal privacy protection and wider spatial sample coverage, an internet-based and self-administered questionnaire was used to obtain voluntary response samples in each sample school from November 2019 to February 2020. College students were asked to answer questions on knowledge, attitudes, and practices (KAP) regarding sexuality, as well as their social demographic characteristics. The survey received 54,580 completed responses in total. Respondents who entered our final analyses were limited to those (1) who provided informed consent, (2) were formally registered students at a Chinese university or vocational college, (3) passed the consistency and quality-control checks, and (4) had a sexual debut, which was defined here as having one’s first incident of penile–vaginal intercourse. To ensure that we could examine the effect of sexual activity on SAs, we excluded those for which suicide occurred before sexual debut from the sample. After applying these inclusion criteria, individual data of 9131 college students were used in the present analysis. To ensure proportional representation of Chinese college students, the final sample was weighted according to the Educational Statistics Yearbook of China 2018, considering the region of educational institution (eastern/central/western), type of educational institution (vocational college/university), grade, and sex.

### 2.2. Exposure

The main exposure of this study was the age at sexual debut. Questions were derived from The Youth Risk Behavior Surveillance System (YRBSS) [33], an epidemiologic surveillance system established by the Centers for Disease Control and Prevention (CDC) and used to conduct cross-sectional analyses. All the participants were asked the following question: “Have you ever had sexual intercourse?”. Then we asked those who answered “yes” a further question: “How old were you when you had sexual intercourse for the first time?”. The age at sexual debut was categorized into 3 groups: “age ≤15 ”, “age 15–18”, and “age ≥ 18” for the following reasons. (1) Early sexual initiation is defined as sexual debut occurring at or before 15. (2) In China, it takes an average of 15 years for a student to earn a junior high school diploma and 18 years to graduate with a senior high school diploma. (3) Because of the huge competitive pressure brought by the national college entrance exam, teachers and parents are strict in supervising adolescents. Thus, sexual behavior is strongly discouraged in senior high school. However, after entering college, most students have reached adulthood (aged 18) at this time, and they are given greater autonomy to make their own decision, so engaging in sexual intercourse is becoming relatively more acceptable than before.

### 2.3. Outcome

The main outcome of this study was SAs. It was assessed using the Suicidality Module of the World Health Organization (WHO) Composite International Diagnostic Interview (CIDI) Version 3.0 [34], which has been used in the National Comorbidity Survey Replication (NCS-R), a nationally representative survey of the US household population [35]. The current study used two items inquiring about lifetime SAs: “Have you ever attempted suicide?” and “How old were you the first time you attempted suicide?”. The second item inquired about only those who disclosed their SA history.

### 2.4. Stratification

Forced sexual debut experience was defined as the first sexual relationship against the will of the individual, which meant being sexually abused to experience first sexual activity by their sexual partner or another person at that time. This was measured with the question: “Were you willing to have a sexual debut?” The five response options were: “Very unwilling”; “Moderately unwilling”; “Neutral”; “Moderately willing”; “Very willing.” For comparison purposes, “Very willing” and “Moderately willing” were considered voluntary, and the remaining groups were considered forced. 

Area of residence was defined as the area of residence before attending university, which was categorized into “urban areas” and “rural areas”. 

Parent–child communication about sex was measured by the question: “How often do your parents talk to you about sexuality?”. The response options were: “Never”; “Rarely”; “Sometimes”; “Frequently”; “Always.” Then responses were dichotomized into “ever” or “never”.

### 2.5. Other Covariates

Age, area of residence, school type, maternal educational level, relationship with mother, family economic status, smoking consumption, alcohol consumption, and sexual orientation were considered as socio-demographic and behavioral covariates. Age was divided into three groups: ≤18, 19–20, and ≥21. Area of residence was categorized into “urban areas” and “rural areas”. School type was categorized into “university” and “vocational college”. Maternal educational level was categorized into “primary school or below”, “junior or senior high school”, “college and above”, and “unknown”. Relationship with mother was assessed using a 10-point self-appraisal scale, and higher scores indicated a better relationship with one’s mother. Family economic status was assessed using a 7-point self-appraisal scale, and higher scores indicated better family financial status. Then we assessed “poor” as scoring from 1–3, 4 illustrated “medium,” and 5–7 meant “rich”. Smoking and alcohol consumption were assessed by asking respondents about the frequency of such activities. Then they were categorized into “yes” and “no”. Sexual orientation was categorized into “heterosexual”, “homosexual”, “bisexual”, and “others”. 

### 2.6. Statistical Analysis

The continuous variable was reported as the mean and standard deviation (SD) while classified variables were described as percentages. Differences in socio-demographic characteristics were assessed using one-way ANOVA (for the continuous variable) or Chi-square tests (for classified variables). Multiple logistic regression was used to assess the associations between SAs and age at sexual debut stratified by sex, forced sexual debut experience, area of residence, and parent–child communication about sex, respectively. OR and 95% CI were calculated to indicate the extent of association between the exposure and outcome variables. Regression models were commonly adjusted for age, area of residence, school type, maternal educational level, relationship with mother, family economic status, smoking consumption, alcohol consumption, and sexual orientation. A two-sided *P* value of less than 0.05 indicated significant differences. All data analyses were conducted using STATA 15.

## 3. Results

Table 1 shows the basic socio-demographic characteristics of participants by sex and age at sexual debut. Among 9131 participants who reported having had a sexual debut, 5051 were female. The proportions of female respondents aged ≤ 18, aged from 19 to 20, and aged ≥ 21 were 6.7%, 49.0%, and 44.4% respectively. The percentages of male respondents aged ≤ 18, aged from 19 to 20, and aged ≥ 21 were 7.0%, 47.4%, and 45.6%, respectively. Having had a sexual debut was reported by 45.8% of rural females and 44.5% of rural males reported having had a sexual debut. In vocational college, 37.6% of females and 43.2% of males reported having had a sexual debut. Most females having had a sexual debut were from a family at a medium economic level, and most males who had a sexual debut were from rich families. The majority of our participants were heterosexual (females: 76.1%, males: 76.7%). Among them, 15.9% of females and 4.4% of males reported experiencing a forced sexual debut. Females appeared to have more parent–child conversations about sex compared to males. 50.1% of females and 46.9% of males had ever had a parent–child conversation about sex. 

Table 2 presents the prevalence of SAs in the study population. Among 9131 participants, 2.4% of females and 1.7% of males had SAs. Females who lived in urban areas (3.2% vs. 1.5%, *p* < 0.05) were more inclined to have SAs. The proportion of having SAs among females in both vocational college and university was higher than that of males. Female students with SAs were in vocational colleges and universities at rates of 2.6% and 2.3%, respectively. Most females who reported SAs were from a poor family; conversely, most males who reported SAs were from a rich family. Both females and males who smoked reported a higher number of SAs compared with those who never smoked. Similarly, both females and males who reported drinking consumption had a higher rate of SAs compared with those who did not. Most females who reported SAs were of other sexual orientations (4.8%) and most males who reported of SAs were bisexual (11.6%). Most participants who reported SAs had a forced sexual debut (females: 3.4%, males: 2.1%). The majority of participants who reported SAs had at least one conversation with their parents about sex (females: 3.2%, males: 2.3%). The proportion of SAs was the highest among females and males who had a sexual debut at an age ≤ 15 (females: 18.7%, males: 5.9%), compared with those who had sex at an older age (females 15–18: 4.6%, females ≥ 18: 0.7%, *p* < 0.001; males 15–18: 5.4%, males ≥18: 0.5%, *p* < 0.001).

Adjusted logistic regression models (Table 3) showed the relationships between having SAs and having a sexual debut at an early age under different circumstances (voluntary or forced sexual debut). Both females and males having experienced sexual debut under age 18 were more likely to report SAs, especially those initiated at the age of 15 or even younger. Regarding forced sexual debut, young females who initiated sex under age 15 were more likely to have SAs (OR 17.04, 95% CI 4.87–59.66, *p* < 0.001); this significant association was only observed in females. Regarding voluntary sexual debut, both females and males were more inclined to have SAs compared to those who initiated sex above or equal to age 18 (females: OR 37.63, 95% CI 14.96–94.66, *p* < 0.001; males: OR 16.19, 95% CI 4.42–59.23, *p* < 0.001). 

In Table 4, multiple regression presents the associations between SAs and age at sexual debut by sex, area of residence, and parent–child communication about sex. Regarding the area of residence, participants in rural areas who had an early sexual debut were more inclined to report having SAs compared to those in urban areas. Among them, females who lived in rural areas and who initiated sexual debut at the age of 15 or even younger were highly likely to report having SAs compared to males who lived in rural areas (females: OR 65.76, 95% CI 19.80–218.42, *p* < 0.001; males: OR 15.39, 95% CI 1.64–144.19, *p* < 0.05). Regarding the frequency of having parent–child communication about sex, respondents who never had such communication, having initiated sexual debut at an early age, were more likely to report having SAs (females: OR 37.81, 95% CI 12.28–116.46, *p* < 0.001; males: OR 11.11, 95% CI 2.62–47.12, *p* < 0.001).

## 4. Discussion

Our analyses showed that young people who reported a sexual debut before the age of 18 were significantly more likely than those reporting a sexual debut after age 18 to report SAs. More importantly, when sexual debut occurred before 15 years old, the experience was associated with a higher risk of SAs. Stratified analyses highlighted that adolescent girls who initiated sex at an early age appear particularly vulnerable to SAs compared to adolescent boys who did so.

Our findings are consistent with those of previous studies. According to an American study of young people aged ≥ 18 years, respondents who reported an age at first sexual intercourse between 12 and 14 years had a 1.46-fold likelihood of SAs compared to those reporting sexual initiation between the ages of 15–17. However, gender differences were not found in the results [10]. A study from South Korea indicated that preteen initiation (first to sixth grades or preschool) of sexual intercourse was significantly associated with an elevated likelihood of SAs compared to teen initiation (seventh to twelfth grades) in both genders [30]. Besides, another study in the United States, using a sample of adolescent girls in 8th–12th grades, highlighted that the younger the females were when they first had sexual intercourse, the greater the number of other problematic behaviors were observed, including having more SAs [29].

The positive association between forced sexual debut and initiation of SAs was consistent with previous studies conducted in both developed and developing countries. Some studies found that both genders in the forced group more often reported adverse reactions (ill health, depression) and psychosomatic symptoms (anxiety, irritation, headaches, and stress) [36,37,38,39], whereas they did not focus on results concerning the relationship between young people who had a voluntary sexual debut experience at an early age and having SAs [23,24]. In our study, we found that young people with early-age voluntary sexual initiations were more likely to report SAs. What is more, those who initiated an early sexual debut under voluntary circumstances were much more likely to have SAs compared to those who did so under forced circumstances, which was not as expected as the conventional mechanisms considering that they were more inclined to have SAs because of the tremendous adverse mental effects resulting from the forced sexual debut experience [23,24]. Thus, it is necessary to explore the potential mechanism of the association between early sexual initiation and having SAs. 

A possible explanation is that young people at adolescence are exactly in a stage when they tend to have a more objective incapacity to meet the demands of rational decision making as well as emotional impulse controlling, which consequently lead to the immature decision of having a sexual debut which accelerates the formulation of SAs [40]. By adolescence, brain regions responsible for primary functions (i.e., sensory and motor) have fully matured, while systems responsible for higher-order functions are still developing [20], especially for the part associated with impulse control, which has not fully matured in adolescence and will not until early adulthood [20,21,22]. This disparity in development makes adolescence a particularly vulnerable period of increased emotional reactivity and risk-taking behaviors [41,42], as well as more emotional dysregulation and impulsivity. Impulsivity is expected to be positively associated with early sexual initiation [43]. At this time, they tend to make quick sexual decisions without devoting much thought to the associated consequences. Additionally, young people aged ≤ 15 were much younger and much more immature compared with other adolescence stages; therefore, according to one study, those who engaged in voluntary sexual activity before age 15 were more likely to have increased depression and anxiety symptoms [44], which may increase the possibility of having SAs. 

In our study, we also found that the odds of SAs were significantly higher in rural areas and for the adolescents who had never had parent–child communication about sex, especially when the sexual debuts occurred under the age of 15. Area of residence and parent–child communication about sex represent the sexual conservatism and disapproving attitudes towards adolescent sexuality in Chinese culture. Thus, those who had early sexual initiations were more likely to gain shame and feelings of exclusion since it is uncommon for adolescents to have a sexual debut before high school graduation in China [45]. When facing problems related to relationship disruption or adverse sexual and reproductive health outcomes, including unintended pregnancy and sexually transmitted infections, they tended to be hesitant to access available support from their family, which may accelerate the production of social isolation. Social isolation was regarded as one of the largest contributors to suicidal risk [46]. Additionally, a mechanism central to the interpersonal-psychological theory of suicide is thwarted belongingness, explained as alienation from friends and family [47,48], which could explain why those who initiated sexual debuts at an early age were more inclined to have SAs for not feeling like a part of a community. 

Regarding those who had sexual debut at younger than age 15, it could be witnessed that female adolescents experience a worse outcome than boys in a conservative area of residence and conservative families, which may result from the conventional Chinese sexual double-standard [2,7]; in male-dominated societies, female sexuality is controlled more tightly [49]. Research on sexual concepts among university students demonstrated that spouse chastity is more important to males than females [50]. Under this context, early sexual initiation leads to a heavier psychological pressure for adolescent girls. A former study found that 40.6% of females felt regret and unease after sexual debut, 29.1% had a sense of loss, and 26.1% felt guilty in a sample of Chinese college students [51]. Thus, the effect of early sexual initiation on their subsequent mental health needs to be paid attention to.

The findings of this study have important implications for educators and prevention scientists who work with adolescents in school or other settings. Since the early onset of sexual activity is commonly associated with adverse outcomes, future sexuality education programs should begin at an earlier age, teaching adolescents to first think about the risks and consequences before engaging in sexual intercourse. These programs should also focus on enhancing adolescents’ skills and control in decision-making on early sexual activity. Variability in the prevalence across regions highlights the need for prevention programs to address the contextual contributing factors. Early sexual initiators, especially girls, should be screened and monitored for suicidal thoughts and behaviors. Given the significant influence of parental communication on children’s sexual behaviors, more work is needed for parents to understand that correct guidance on sexual issues could be a better way than avoiding talking or blindly opposing. They need to improve their communication skills and strive to provide a supportive environment for their children, to better instruct them and protect against any risks related to sexual behaviors. Our study also suggests that adolescent sexual behaviors should be considered in the context of cultural factors, which may inspire more research to be conducted to better understand the social-sexual development of Chinese young people.

Several limitations should be considered when interpreting our main findings. Firstly, causality could not be directly established because of the cross-sectional study design. Thus, further prospective research is expected to be undertaken to assess the causality of such an association. Secondly, this study involved retrospective self-reports of age at sexual debut, age at first SA, and other information by participants, which may have introduced recall bias and/or inaccurate reporting into the results. Thirdly, the measures of sexual debut and SAs were both based on two items. Given the complex nature of sexual behavior and suicidality, future research should expand the number of questions asked related to these variables. Fourthly, as the population was limited to university and college students, the generalization of our findings to other adolescent and youth populations should be made with caution.

NCSS-SRH is the largest and most comprehensive survey on the sexual and reproductive health of college students in China so far. Because of a balanced spatial distribution of public HEIs across China, the respondents have a relatively broad geographical distribution in the eastern, central, and western regions. They cover 31 provincial administrative regions, which may reduce sampling error and increase the national representativeness of the sample. This is the first study to examine the association between early sexual debut and SAs among Chinese college students. The results from our study bridge the knowledge gap and provide key public health information about this population. Our study also extended the existing literature on the association between sexual debut and SAs further by considering the classification of forced or voluntary sexual initiation and the impact of Chinese social norms on sex.

## 5. Conclusions

In conclusion, we found that early sexual initiation was associated with later SAs among Chinese young people, especially girls. This association was positive in both the forced debut and voluntary debut groups. Increased risks of SAs were reported in rural areas and from the adolescents who had never had parent–child communication about sex. These findings provide useful evidence for applying interventions to reduce the adverse psychological outcomes caused by early sexual initiation. Interventions targeting adolescents should include comprehensive, timely, and gender-sensitive sexuality education as well as liberal and supportive parental communication about sex.

## Figures and Tables

**Table 1:** Basic characteristics of participants by sex and age at sexual debut (N = 9131).

Characteristics	Female					Male				
	Total	≤15	15–18	≥18	*p*-Value	Total	≤15	15–18	≥18	*p*-Value
N ^a^ (% ^b^)	5051	268 (5.9)	955 (17.0)	3828 (77.1)		4080	300 (5.0)	1131 (18.8)	2649 (76.2)	
Age (% ^b^)					<0.001					<0.001
≤18	6.7	16.3	14.9	4.1		7.0	19.8	18.2	3.4	
19–20	49.0	43.0	59.1	47.2		47.4	46.7	52.4	46.2	
≥21	44.4	40.8	26.0	48.7		45.6	33.5	29.4	50.4	
Area of residence (% ^b^)					0.385					0.702
Urban	54.2	58.6	58.0	53.0		55.5	40.5	43.3	45.1	
Rural	45.8	41.4	42.1	47.0		44.5	59.5	56.7	55.0	
School type (% ^b^)					<0.001					0.004
Vocational college	37.6	53.0	46.2	34.5		43.2	38.9	53.5	41.0	
University	62.4	47.0	53.8	65.5		56.8	61.2	46.5	59.0	
Maternal educational level (% ^b^)					0.015					0.002
Primary school or below	26.7	29.3	21.5	27.7		25.8	21.7	18.0	28.0	
Middle or high school	53.0	51.9	56.0	52.5		55.0	52.2	63.4	53.0	
College and above	19.3	14.3	20.8	19.4		18.4	24.8	17.7	18.2	
Unknown	1.0	4.6	1.7	0.5		0.8	0.8	0.9	0.8	
Relationship with mother ^c^ (Mean ± SD ^b^)	7.09 ± 2.21	7.05 ± 2.23	6.87 ± 2.35	7.14 ± 2.17	0.005	7.92 ± 1.90	7.54 ± 2.15	7.79 ± 1.92	7.98 ± 1.87	<0.001
Family economic status (% ^b^)					0.803					0.300
Poor	28.9	28.5	32.4	28.2		25.7	27.0	21.8	26.6	
Medium	37.8	40.6	36.6	37.8		37.0	41.8	36.2	36.8	
Rich	33.3	30.9	31.0	34.1		37.3	31.2	41.9	36.6	
Smoking consumption (% ^b^)					<0.001					<0.001
Yes	21.0	37.1	38.5	15.9		43.7	55.8	60.1	38.8	
No	79.0	62.9	61.5	84.1		56.3	44.2	39.9	61.2	
Alcohol consumption (% ^b^)					0.001					0.001
Yes	51.0	61.1	61.7	47.9		66.8	73.1	75.8	64.2	
No	49.0	38.9	38.4	52.1		33.2	26.9	24.2	35.9	
Sexual orientation (% ^b^)					0.014					<0.001
Heterosexual	76.1	74.1	71.4	77.3		76.7	60.2	75.5	78.1	
Homosexual	25.8	3.6	4.6	2.1		13.5	24.5	9.5	13.7	
Bisexual	16.5	19.7	20.2	15.5		7.1	6.4	12.0	5.9	
Others	4.8	2.6	3.8	5.2		2.7	8.9	3.0	2.3	
Forced sexual debut (% ^b^)					<0.001					0.035
Yes	15.9	29.2	26.3	12.6		4.4	10.2	4.2	4.0	
No	84.1	70.8	73.8	87.4		95.7	89.8	95.8	96.0	
Parent–child communication about sex					0.526					0.792
Ever	50.1	57.0	49.0	49.8		46.9	46.9	48.7	46.5	
Never	49.9	43.0	51.0	50.2		53.1	53.1	51.3	53.5	

Note. ^a^ Unweighted. ^b^ Weighted. ^c^ Relationship with mother was assessed with a 10-point self-assessment scale, with higher scores indicating a better relationship with one’s mother.

**Table 2:** Prevalence of SAs by sex (N = 9131).

Characteristics	Female			Male		
	N ^a^	N ^a^ (% ^b^) Who Had SA	*p*-Value	N ^a^	N ^a^ (% ^b^) Who Had SA	*p*-Value
N ^a^ (% ^b^)	5051	131 (2.4)		4080	59 (1.7)	
Age			0.001			0.067
≤18	829	32 (10.0)		871	18 (2.4)	
19–20	2660	65 (1.8)		2110	24 (0.7)	
≥21	1562	34 (2.0)		1099	17 (2.6)	
Area of residence			0.040			0.131
Urban	2869	98 (3.2)		2235	37 (1.0)	
Rural	2182	33 (1.5)		1845	22 (2.6)	
School type			0.804			0.362
Vocational college	1105	35 (2.6)		1514	27 (2.3)	
University	3946	96 (2.3)		2566	32 (1.2)	
Maternal educational level			0.39			0.853
Primary school or below	1180	19 (1.6)		1038	14 (1.5)	
Middle or high school	2629	73 (2.9)		2148	25 (1.9)	
College and above	1198	38 (2.4)		840	20 (1.5)	
Unknown	44	1 (1.5)		54	0 (0.0)	
Relationship with mother ^c^ (Mean ± SD ^b^)	6.80 ± 2.51		0.137	7.11 ± 2.01		<0.001
Family economic status			0.963			0.326
Poor	1239	38 (2.6)		1025	13 (0.8)	
Medium	1876	54 (2.4)		1460	22 (1.5)	
Rich	1936	39 (2.4)		1595	24 (2.6)	
Smoking consumption			<0.001			0.311
Yes	1012	79 (6.8)		1868	33 (2.4)	
No	4039	52 (1.3)		2212	26 (1.2)	
Alcohol consumption			0.110			0.592
Yes	2532	100 (3.4)		2842	39 (1.9)	
No	2519	31 (1.4)		1238	20 (1.3)	
Sexual orientation			0.036			<0.001
Heterosexual	3782	76 (2.0)		3306	33 (0.6)	
Homosexual	137	3 (1.4)		410	13 (2.4)	
Bisexual	860	42 (4.1)		229	9 (11.6)	
Others	272	10 (4.8)		135	4 (3.1)	
Forced sexual debut			0.228			0.741
Yes	774	41 (3.4)		161	6 (2.1)	
No	4277	90 (2.3)		3919	53 (1.7)	
Parent–child communication about sex			0.072			0.346
Ever	2636	82 (3.2)		1860	27 (2.3)	
Never	2415	49 (1.7)		2220	32 (1.2)	
Age at sexual debut			<0.001			<0.001
≤15	268	39 (18.7)		300	16 (5.9)	
15–18	955	56 (4.6)		1131	25 (5.4)	
≥18	3828	36 (0.7)		2649	18 (0.5)	

Note. ^a^ Unweighted. ^b^ Weighted. ^c^ Relationship with mother was assessed with a 10-point self-assessment scale, with higher scores indicating a better relationship with one’s mother.

**Table 3:** The associations between SAs and age at sexual debut by sex and forced sexual debut experience.

	Forced Sexual Debut	Voluntary Sexual Debut
	OR (95% CI)	OR (95% CI)
	Female (N = 5051)	
Age at sexual debut		
≥18	1.00 (Ref)	1.00 (Ref)
15–18	10.38 (2.64, 40.82) ***	2.95 (1.45, 6.03) **
≤15	17.04 (4.87, 59.66) ***	37.63 (14.96, 94.66) ***
	Male (N = 4080)	
Age at sexual debut		
≥18	1.00 (Ref)	1.00 (Ref)
15–18	11.53 (0.60, 222.42)	9.06 (3.46, 23.74) ***
≤15	3.70 (0.48, 28.24)	16.19 (4.42, 59.23) ***

Note. Adjusted for: age, area of residence, school type, maternal educational level, relationship with mother, family economic status, smoking consumption, alcohol consumption, and sexual orientation. ** *p* < 0.01, *** *p* < 0.001.

**Table 4:** The associations between SAs and age at sexual debut by sex, area of residence, and parent–child communication about sex.

	Area of Residence ^a^		Parent–Child Conversations about Sex (frequency) ^b^	
	Urban	Rural	Ever	Never
	Female (N = 5051)			
Age at sexual debut				
≥18	1.00 (Ref)	1.00 (Ref)	1.00 (Ref)	1.00 (Ref)
15–18	3.43 (1.77, 6.66) ^***^	10.65 (3.36, 33.74) ^***^	3.09 (1.37, 6.96) ^**^	8.36 (3.00, 23.29) ^***^
≤15	19.86 (7.82, 50.44) ^***^	65.76 (19.80, 218.42) ^***^	21.81 (9.01, 52.77) ^***^	37.81 (12.28, 116.46) ^***^
	Male (N= 4080)			
Age at sexual debut				
≥18	1.00 (Ref)	1.00 (Ref)	1.00 (Ref)	1.00 (Ref)
15–18	7.33 (2.23, 24.03) ^***^	7.52 (2.41, 23.47) ^***^	7.17 (3.09, 16.66) ^***^	3.29 (0.99, 10.98)
≤15	10.73 (2.89, 39.88) ^***^	15.39 (1.64, 144.19) ^*^	3.18 (0.44, 22.98)	11.11 (2.62, 47.12) ^***^

Note. ^a^ Adjusted for: age, school type, maternal educational level, relationship with mother, family economic status, smoking consumption, alcohol consumption, and sexual orientation. ^b^ Adjusted for: age, area of residence, school type, maternal educational level, relationship with mother, family economic status, smoking consumption, alcohol consumption, and sexual orientation. * *p* < 0.05, ** *p* < 0.01, *** *p* < 0.001.

## Data Availability

The datasets generated during and/or analyzed during the current study are not publicly available due to conditions on participant consent and other ethical restrictions.
